# Older adults’ participation in artistic activities: a scoping review

**DOI:** 10.1007/s10433-022-00708-z

**Published:** 2022-05-26

**Authors:** Karima Chacur, Rodrigo Serrat, Feliciano Villar

**Affiliations:** Department of Cognition, Development and Educational Psychology, University of Barcelona, Passeig de la Vall d’Hebron 171, 08035 Barcelona, Spain

**Keywords:** Participation in artistic activities, Older people, Active aging, Scoping review

## Abstract

This scoping review analyses existing literature on older adults’ participation in artistic activities. It identifies gaps in this research topic and suggests new directions for research. We followed the five-step process defined by Arksey and O’Malley (2005) and extended by Levac et al. (2010). Four electronic databases were searched, and 129 peer-reviewed articles were included in the scoping review. Research into older adults’ participation in artistic activities has grown in the last ten years. However, empirical papers tend to focus on the outcomes of older people’s participation in artistic activities, in particular the benefits. Most papers centred on facilitators to examine the antecedents of this type of participation among people in late life. Research about experiences, potentially negative consequences or barriers to older adults’ participation in artistic activities have been largely overlooked. We identified several gaps in the literature, which we classified as: related to the artistic activities that were considered; the potential costs and barriers for older adults’ participation in artistic activities; older adults’ voices and their diversity; the life course perspective; and a contextual view of research on the topic. These gaps suggest challenges that future research on older adults’ participation in artistic activities should consider.

## Introduction

In recent decades, a positive perspective on aging has emerged that has questioned the traditional view of later life as a period only characterized by deficit and decline. The concept of active aging is one of the most influential of those proposed to understand positive aspects of aging [World Health Organization (WHO) 2002]. Active aging alludes to “the process of optimizing opportunities for health, participation and security in order to enhance quality of life as people age” (WHO 2002, p. 12). The concept of active aging brings to the fore older adults’ potential for realizing their own physical, social and mental well-being, with a focus on mainstreaming participation and health over the life course (WHO 2002). A key aspect for defining and promoting active aging is to identify which activities are included in the concept (Boudiny 2013). A broad range of activities has been proposed as epitomes of active aging, such as paid work (e.g., Zacher et al. 2018), physical activity (e.g., Bauman et al. 2016) and volunteering (e.g., Lie et al. 2009).

However, other activities that could potentially contribute to aging well have been much less explored. Among these, older adults’ participation in artistic activities deserves special attention, as it has been associated with several individual benefits, such as better health (e.g., Cohen 2006), greater well-being (e.g., Creech et al. 2013), and higher quality of life (e.g., Johnson et al. 2017). Participation in artistic activities in later life has also been related to a number of social benefits, such as maintaining close social networks during this life period (e.g., Jeffri 2005). Interventions focused on artistic activities for therapeutic and non-therapeutic purposes for older people (e.g., Gutheil and Heyman 2016; Dunphy et al. 2019) have been widely studied. Nevertheless, there is far less research on older people’s spontaneous, voluntary participation in artistic activities. The term “spontaneous participation” refers to voluntary engagement in artistic activities that do not have research, educational, or therapeutic objectives. The objective of this study was to fill that gap and revise current research on this area. The revision was designed to identify critical areas that need further research development.

### What do we know about older adults’ participation in artistic activities?

Research on older adults’ participation in artistic activities has focused on a variety of arts disciplines, such as music (Johnson et al. 2017), visual arts (Lindauer et al. 1997), theatre (Bernard et al. 2015), crafts (Maidment and Macfarlane 2011), literature (Swinnen 2018) or dance (Wainwright and Turner 2004). This line of research has examined amateur (Liddle et al. 2014) and professional levels (Henoch and Chesky 1999), as well as individual (Li 2012) and group participation in artistic activities (Wise et al. 1992). Previous research into older adults’ participation in artistic activities could be broadly classified as considering the role of the activity in empirical studies. Some studies explored factors that may promote participation in artistic activities in later life (Fisher and Specht 1999), to ascertain the antecedents of this participation. These antecedents could be classified as individual, interpersonal or environmental. In relation to individual factors, motivations for participation have been studied including intrinsic motivations among professional visual artists, who perceived an inherent need for self-expression and self-actualization (Reed 2005). In a similar way, Fisher and Specht (1999) detected a strong need to create among older participants at a senior art exhibition. Interpersonal aspects have also been explored, such as family influences in the development of an artistic career, and it has been found that almost all professional classical musicians came from families with some musical practices (Manturzewska 1990). The effect of a partner’s support on older women’s participation in artistic activities at amateur level has also been studied. Reynolds (2009) reported that partners could play a significant role in inspiring women’s artistic activity, both through giving support and through recognizing their need to have some separate interests. These interpersonal influences on artistic participation can be understood through a life course approach, under the concept of “linked lives” developed by Elder (Elder 1998; Bengtson et al. 2005), which highlights the interconnectedness of the lives of different people. In this regard, lives —including artists’ lives— are rooted in relationships with other individuals and are therefore influenced by them. Finally, regarding environmental antecedents, some studies have focused on context-related barriers that hinder participation. For example, absence of transport could limit older people’s artistic participation in leisure activities (Keaney and Oskala 2007).

In contrast to research focused on antecedents, other studies have addressed the consequences of older adults’ participation in artistic activities (Liddle et al. 2012). That is, the focus is the outcomes of these activities. Artistic activities have been associated, at individual level, with an improvement in physical health (Reynolds et al. 2011), better cognitive function (Gray and Gow 2020; Mansens et al. 2018) and greater well-being (Kenning 2015). Besides, artistic participation has been related with a positive impact on successful aging, a concept that comprises various dimensions, such as relations with people, sense of purpose, autonomy, personal growth, self-acceptance (Schofield-Tomschin and Littrell 2001), and an attitudinal orientation to life in general (Fisher and Specht 1999). Regarding individual outcomes of artistic activities according to the level of artists’ expertise, artistic participation had a positive effect on the well-being of both professional and amateur artists (Swinnen 2019). However, certain consequences were detected only in professional artists, such as the feeling of fulfilment, which was linked more to the process of artistic creation than to recognition by other artists and clients (Matarasso 2012).

In addition to the examination of individual benefits, some studies have focused on the social benefits of participating in artistic activities, such as mutual support in members of a male voice choir (Reynolds 2015), decrease of isolation in older participants in a women’s community choir (Southcott and Joseph 2015) or transmission of cultural heritage to others by members of community singing groups (Joseph and Southcott 2018). In contrast to benefit-focused studies, few investigations have centred on the negative consequences of participation in artistic activities. An example is the study by Barbeau and Mantie (2019) on music performance anxiety, which found that musicians were worried about matters related to novelty, unpredictability, and the absence of control in settings of public performances.

Finally, other studies examined the experience of participating in artistic activities. For instance, the subjective experiences and meanings connected with this participation have been explored in amateur artists. The study by Pöllänen (2013) revealed that craft-making strengthened the sense of meaningfulness and that artistic products were tangible symbols of life experiences. The role of musical participation in identity processes has also been studied, in the sense of the re-shaping of identity, by means of the re-identification of orchestra participants as musicians (Jenkins and Southcott 2016).

Regardless of the focus of studies, social gerontologists have generally attempted to promote an age-integrated society and recognize the diversity of aging processes (Hooyman and Kiyak 2011). Older adults’ diversity is a relevant aspect of research on participation in artistic activities, as such diversity could shape aspects such as who participates in these activities and in which ways. To date, there have been some studies on older women’s (e.g., McHugh 2016) or older migrants’ (e.g., Li 2012) participation in artistic activities.

Notwithstanding the growing interest of researchers in older adults’ participation in artistic activities, there are no comprehensive, exhaustive reviews on this topic. To the best of our knowledge, the only review conducted to date is that by Carr et al. (2009) about “arts and aging research”. However, this review was conducted over ten years ago and only screened four gerontological journals, without incorporating a further broad database exploration. Moreover, a relatively small amount of the literature reviewed describes older adults’ spontaneous and volunteer participation in artistic activities. For the reasons presented previously, the present scoping review sought to examine the development of research on older people’s participation in artistic activities and to explore which aspects of this participation have been considered to date. Additionally, we aimed to identify critical gaps and future lines of research in older adults’ participation in artistic activities.

## Method

The present study is a scoping review about older people’s participation in artistic activities. Scoping studies are especially useful for exploring a wide range of literature and identifying research gaps (Arksey and O’Malley 2005). In our study, we followed the five scoping review phases developed by Arksey and O’Malley (2005), expanded by Levac et al. (2010). As suggested by Munn et al. (2018), our scoping review also sought to identify the key concepts and definitions related to older people’s artistic participation, as well as their main characteristics.

### Step 1: Determine the purpose

The aim of this scoping review was to describe the magnitude and nature of published studies on older adults’ spontaneous, voluntary participation in artistic activities. The purpose was also to identify knowledge gaps on this issue, as well as to identify key concepts and definitions related to older people’s artistic participation. For our study, participation in artistic activities was understood as the active, spontaneous practice of creative arts, including visual arts, literature, dance, music, theatre, crafts and cinema, among other types of artistic activity. We defined older people as people aged 50 or over, or when information on age was not available, people identified as old by the authors of the publication.

### Step 2: Identify potential studies

We developed search strategies with a professional librarian, and search terms and databases were identified through an iterative process. Four databases were searched to identify potential studies in March 2020. The databases included PsycINFO, Sociological Abstracts, Web of Science and Scopus. The search strategy used keywords related to participation in artistic activities and older people (see Table 1). Articles were included if they were peer-reviewed and written in English, with no date limitations.

**Table 1:** Keyword’s combinations used in the Scoping review

(Ageing OR aging OR old age OR older* OR seniors OR senior citizens OR elder* OR later life OR third age OR aged people OR aged persons OR aged men OR aged women OR centenarians OR life course)	AND	(Arts OR cultural OR creative OR art OR artistic OR painting OR literary OR performance OR musical OR theatre OR drama OR craft AND activity)
		(Cultural OR creative OR art OR artistic OR painting OR literary OR performance OR musical OR theatre OR drama OR craft AND activities)
		(Cultural OR creative OR art OR artistic OR painting OR literary OR performance OR musical OR theatre OR drama OR craft AND project)
		(Cultural OR creative OR art OR artistic OR painting OR literary OR performance OR musical OR theatre OR drama OR craft AND program)
		(Creative OR cultural OR musical OR artistic AND engagement)
		(Creative OR cultural OR musical OR artistic AND participation)
		(Creative OR cultural OR musical OR artistic AND involvement)
		(Musical OR theatre AND performer)
		(Artistic OR creative AND mediums)
		(Art-making OR artistic creativity OR professional musician OR artist OR artists OR art practice)

### Step 3: Screen and select relevant studies

We used a standardized form to select the relevant studies. For this purpose, we applied the following two inclusion criteria: the paper was centred on older adults, and the focus of the study was participation in artistic activities. Regarding the first inclusion criterion, papers that were not on older adults were excluded, as were papers that presented older and younger people, and the results were not compared. For the second inclusion criterion of studies focused on artistic activity, papers had to have the following characteristics: artistic activity was necessarily a primary focus of the study (studies with a broader focus were excluded); the artistic activity was spontaneously/voluntarily practiced (studies about artistic practices carried out specifically for the research, or in the format of an intervention, project, programme, workshop or university class were excluded); the artistic discipline was defined as a creative practice, and not only as physical (for example: if dance was performed mainly for exercise, and not as a creative activity it was not eligible); the artistic activity was defined as an active not a passive practice (for example: passive artistic activities such as visiting an exhibition, seeing a play or listening to music were not included). Finally, articles that were not available online were not incorporated in the review. To decide on the inclusion or exclusion of articles, we reviewed the titles and abstracts. If the abstract did not provide enough information to decide, we proceeded to review the entire text of the papers. When the inclusion/exclusion of a paper was not clear, we reached an agreement through discussion, and the criteria were clarified and revised.

### Step 4: Extract data into charts

Data were extracted from the final sample of papers using a data-charting form in Microsoft Excel (Arksey and O’Malley 2005). A trial extraction was conducted in which we obtained data on 30% of the articles. These preliminary results were discussed, and changes to the data-charting form were made if necessary. In addition to bibliographic information, the data that were extracted included the purpose of the study (or research question), conceptual elements (focus on antecedents, experiences or outcomes; life-span perspective included or excluded), characteristics of the artistic activity (discipline, individual or group practice, professional or amateur practice), methodology (research design, data collection technique, sample characteristics), and key findings and conclusions.

### Step 5: Collate and summarize the results

We reviewed the extracted data to define the best way to summarize the findings and agreed on carrying out two kinds of analyses (Arksey and O’Malley 2005; Levac et al. 2010). Firstly, a descriptive summary was compiled of categories associated with general trends on this research topic (main focus, artistic activity characteristics and main characteristics). Secondly, we conducted a qualitative content analysis of the studies classified under the focus of antecedents, experiences or outcomes.

## Results

**Fig. 1:**
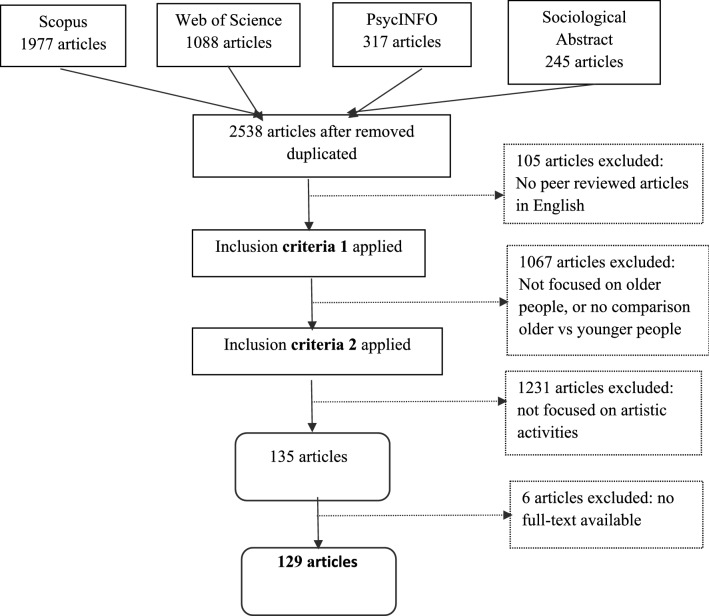
Flow chart. Scoping review on older people’s arts activities

Figure 1 shows the process of selecting the papers.

The analysis of 129 articles is presented in two sections. In the first part, we provide a descriptive summary of the extent, distribution and characteristics of older people’s participation in artistic activities. In the second part, we present the results of a qualitative content analysis of the studies.

### Descriptive summary

#### Expansion and extension of research on older adults’ participation in artistic activities

**Fig. 2:**
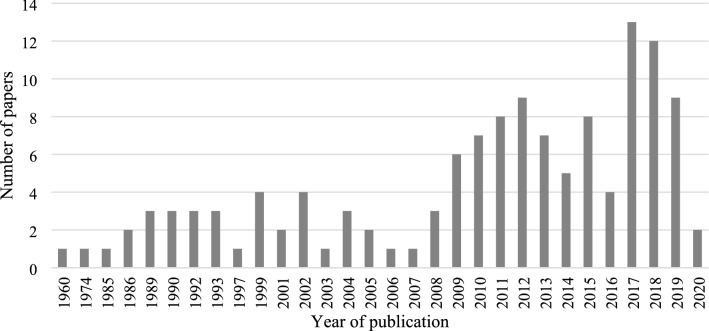
Number of publications on older adult's participation in artistic activities, by year of publication. Period 1960–2020. *N* = 129

The studies included in this scoping review were published between 1960 and 2020 (see Fig. 2). There has been a growing interest in this topic since 2010, with 65.1% (*n* = 84) of the papers published after this year (see Fig. 2).

Papers were classified into review, conceptual and empirical. Eight studies (6.2%) corresponded to review articles. These papers investigated in a broader manner arts and aging research (Carr et al. 2009); examined specific disciplines among older people, such as theatre (Bernard et al. 2017) and music (Coffman 2002; Jordan 2019; Park 2015); or explored specific elements of artistic activities in later life, such as quality of life (Fraser et al. 2015), creativity (Price and Tinker 2014) and benefits (Noice et al. 2014).

Fifty-two papers (40.3%) were classified as conceptual articles. These studies included several themes, such as the analysis of older iconic or eminent creators considering aging elements (Beckerman 1990; Catbagan and Musser 2019; Lindauer 1992; Nuessel et al. 2001; Pritikin 1990), the evolution of creativity linked to artistic activity and the development of creative practice in later life (Bruhn 2002; Cohen-Shalev 1986, 1989; Greenleigh 1960); policies related with arts and aging (Lehikoinen 2017); positive aging through arts (Brodsky 2011); benefits of artistic activities (Wood et al. 2018); and personal artistic experiences (LaBorie 2013; Slater 2019).

Empirical studies made up 53.5% of the total papers that were studied (*n* = 69). According to the place where samples were collected, they were conducted mostly in the United States of America (26.1%; *n* = 18), the United Kingdom (18.7%; *n* = 13) and Australia (15.8%; *n* = 11). Other countries were underrepresented or absent completely, and only one paper met the characteristics of a cross-cultural study (see Table 2). In the following sections, the empirical papers are described in terms of methodology and conceptual elements.

**Table 2:** Country of origin of the sample (empirical papers; N = 69)

Country of the sample	*N*	%
One country	67	97.0
United States	18	26.0
United Kingdom	13	18.7
Australia	11	15.8
Netherlands	3	4.2
Japan	3	4.2
Poland	2	2.9
Finland	2	2.9
Canada	2	2.9
Israel	2	2.9
Italy	1	1.5
Philippines	1	1.5
New Zealand	1	1.5
India	1	1.5
Singapore	1	1.5
Sweden	1	1.5
Austria	1	1.5
Argentina	1	1.5
Greece	1	1.5
Germany	1	1.5
Belgium	1	1.5
More than one country	2	3.0
Not cross-cultural	1	1.5
Cross-cultural	1	1.5
Total	69	100

#### The methodological approach to older adults’ participation in artistic activities

In terms of methodology, slightly more papers used a qualitative design (52.2%) than a quantitative design (42%). Very few studies used mixed methods designs (5.8%). Almost all studies made use of cross-sectional designs (95.7%). In general, studies were centred specifically on older adults (79.7%), with a smaller proportion comparing older with younger people (20.3%). Data of the studies were collected mainly through interviews (39.1%) and questionnaires (31.9%) (see Table 3).

**Table 3:** Empirical papers’ key methodological characteristics (in frequencies and percentages) (*N* = 69)

Methodological aspects	Empirical papers	
	*N*	%
Methodology		
Qualitative	36	(52.2)
Quantitative	29	(42.0)
Mixed methods	4	(5.8)
Research design		
Cross-sectional	66	(95.7)
Longitudinal	3	(4.3)
Data collection		
Interviews	27	(39.1)
Questionnaire	22	(31.9)
Ethnography	5	(7.2)
Focus group	2	(2.9)
More than one	13	(18.9)
Age focus		
Focus on older adults	55	(79.7)
Focus on the comparison with younger age groups	14	(20.3)

#### Conceptual elements of empirical research on older adults’ participation in artistic activities

Empirical papers included a variety of artistic disciplines as the main focus. Most studies examined music (40.6%) and visual arts activities (17.4%). Other artistic disciplines were less represented (see Table 4). In relation to the characteristics of the artistic activities, one-third of the studies (34.8%) examined group practices, while 14.5% of the papers described individual activities. In addition, studies covered both amateur (50.7%) and professional (31.9%) artistic activities (see Table 4).

**Table 4:** Empirical papers’ key conceptual characteristics (in frequencies and percentages) (*N* = 69)

Conceptual aspects	Empirical papers	
	*N*	%
Process model^a^		
Antecedents	23	(33.3)
Outcomes	54	(78.2)
Experiences	17	(24.6)
Type of practice		
Individual	10	(14.5)
Group	24	(34.8)
Both	18	(6.1)
Unclear	17	(24.6)
Artistic level		
Professional	22	(31.9)
Amateur	35	(50.7)
Professional and amateur	11	(15.9)
Unclear	1	(1.5)
Artistic discipline		
One discipline	54	(78.3)
Music	28	(40.6)
Visual arts	12	(17.4)
Crafts	7	(10.1)
Theatre	5	(7.2)
Literature	1	(1.5)
Dance	1	(1.5)
More than one discipline	15	(21.7)
Visual arts and crafts	5	(7.1)
Visual arts and music	1	(1.5)
Theatre and music	1	(1.5)
Three or more disciplines	7	(10.1)
Unclear	1	(1.5)
Life-span perspective		
No	40	(58.0)
Yes	29	(42.0)
Attention to diversity		
Yes	27	(39.1)
Women	9	(13.0)
Racial/ethnic minorities	4	(5.8)
Immigrants	4	(5.8)
Rural	1	(1.5)
Disability/health issues	1	(1.5)
More than one	8	(11.5)
No	42	(60.9)

^a^The sum of categories’ frequencies could exceed the total number of papers as a same paper could be classified in more than one category

Concerning the role of artistic activity in the studies, most empirical papers referred to the outcomes of older people’s arts activities (78.2%). A total of 33.3% of papers addressed antecedents of artistic activities and 24.6% focused on the experiences of artistic participation. Notably, the same paper can be classified in more than one of these categories, because some studies analyzed more than one component, for example antecedents and outcomes (Fisher and Specht 1999) (see Table 4). Finally, diversity (described as a key aspect of research on diverse, possibly relegated groups of older persons) was present in one-third of the studies (39.1%), principally considering older women (13%), older persons who belong to ethnic or racial minorities (5.8%) and older immigrants (5.8%) (see Table 4).

### Thematic analysis

As previously mentioned, research about older people’s participation in artistic activities can be classified into papers focused on antecedents, outcomes or experiences of artistic participation. For this reason, studies distributed according to these three focuses are presented below.

#### Antecedents

Twenty-three papers addressed the antecedents of artistic activity. Studies about antecedents can be categorized as individual, interpersonal or environmental factors that could positively influence or promote artistic participation (facilitators) or adversely influence or hinder older people’s artistic activities (barriers). Research has focused principally on facilitators of artistic participation among older adults, with few studies centred on barriers.

##### Individual level

At individual level, different elements could act as predictors of participation in artistic activities. Sociodemographic aspects, such as a higher socioeconomic status (Keaney and Oskala 2007) could promote amateur artistic participation among older persons. Regarding gender, it was found that in the music profession, even though more women begin musical careers, their number decreases at the end of their professional careers. A possible explanation for this situation might be the differences in motivation, both intrinsic and extrinsic, between women and men (Manturzewska 1990). In terms of age, some studies have shown that artistic participation decreases as a person gets older. However, there are some activities, such as crafts and textiles, that are significantly more popular hobbies in old age than in young adulthood. The fact that crafts activities can be carried out at home facilitates artistic engagement by a greater range of people, since other factors such as transport access or poor health do not intervene (Keaney and Oskala 2007). Pre-existing skills (Reynolds 2009) or good physical health (Liddle et al. 2014) can also promote participation in artistic activities. However, it is not entirely clear whether artistic participation leads to better health or whether being in good health encourages participation, or whether the two are combined (Liddle et al. 2014). The impact of poor health, especially among people with a limiting disability, could restrict engagement in artistic activities. Although this study did not address the reasons for this decline in artistic participation, poor health may clearly act as a barrier (Keaney and Oskala 2007).

Above social or material motivation, interest and personal motivation can considerably influence the continuity of creative activity during aging (Pufal-Struzik 1993). Motivation can play a relevant role in choosing an artistic activity, in a more noticeable way in professional than in amateur artists. It may appear as intrinsic motivation to continue working in the visual arts (Reed 2005) or to carry on developing a music career (Manturzewska 1990). Other intrinsic motivations are related to the need to create or the desire to express the inner self (Fisher and Specht 1999) or as an innate desire or a need to be fulfilled (Mullen et al. 2012).

Life course factors can influence artistic participation. Therefore, some studies focused on how aging affects aspects of arts practices among professional artists and found that creativity increases over the years (Lindauer et al. 1997; Reed 2005). In the study by Lindauer et al. (1997), older artists perceived that the quality and quantity of their artwork had improved over the decades, as well as the sources of their creativity. Nevertheless, other studies suggest that musical performance of professional musicians declines with age. In this research, most musicians believed that peak performance on their musical instrument was reached before 30 years or between the ages of 30 and 39, and that performance starts to decay later (Gembris and Heye 2014). Unwanted experiences during the life course, such as illness, can also facilitate artistic participation as a way of promoting the search for meaningful leisure occupations. In fact, the artistic process can also be influenced by illness, in the sense of a positive effect on both the nature of the artistic theme and the technique used in artworks (Reynolds 2004). However, some transitions during middle adulthood could also act as barriers, particularly among professional artists. One of these is having children, a situation that can lead to temporary abandonment of the artistic career and the choice of careers in non-artistic fields, to favour more economic stability (Mullen et al. 2012).

##### Interpersonal level

Interpersonal aspects have been also studied, for example, family influence on the development of a musical (Manturzewska 1990), performing arts (Mullen et al. 2012) or visual arts career (Reynolds 2009), finding that frequently artists —for instance, musicians— come from families with some artistic tradition (Manturzewska 1990). Similarly, close people can promote artistic participation in later life, for example, through support given by older participants’ partners and sensitive encouragement from friends and relatives (Reynolds 2009). Moving on to the interpersonal elements that may obstruct artistic participation, a lack of social networks could reduce involvement in leisure artistic activities, particularly in the oldest-old persons. Although there is no clear age-related trend regarding lack of company as a barrier, it is an item that is mentioned more by older people than by younger people (Keaney and Oskala 2007).

##### Environmental level

Environmental elements have been studied to a lesser extent. In terms of facilitators, childhood home environment probably has a more relevant influence on visual artists than schooling (Dohr and Forbess 1986). Concerning barriers, a lack of transport could hinder artistic participation as it reduces older people’s access to artistic activities as hobbies (Keaney and Oskala 2007). Other contextual elements, such as economic aspects could hinder the professional careers of performing artists, diverting them towards other non-performing arts careers (Mullen et al. 2012). Finally, social changes such as variations in audiences’ interests could be a great challenge for older professional musicians, and may demand a transformation in their skills (Barton 2004).

#### Outcomes

Fifty-four papers focused on outcomes of older adults’ artistic activities. These outcomes can be categorized according to the benefits and negative effects of artistic activities.

##### Benefits

Benefits is the topic that is most frequently studied in relation to older people’s participation in artistic activities, including studies focused on individual (health, cognition, and emotional aspects) and social benefits. In this sense, numerous individual health benefits have been described, in both professional and amateur artists. For example, making music (Creech et al. 2013), painting (Liddle et al. 2012) and textile craftwork (Kenning 2015) could enhance physical and mental health. Likewise, art-making is positively associated with health-related quality of life and especially with physical capacity and independence (Liddle et al. 2014). In the same way, older choir singers reported higher physical quality of life (Johnson et al. 2017; Joseph and Southcott 2018). In relation to cognitive aspects, musical activity among older persons could enhance several domains, at both professional and amateur level. Therefore, studies have shown that musical participation improves verbal working memory (Hanna-Pladdy and Gajewski 2012), executive processes (Hanna-Pladdy and MacKay 2011), learning and attention (Mansens et al. 2018) and visuo-spatial abilities (Gray and Gow 2020). In the same way, dancing seems to be associated with better cognitive function (Masutani et al. 2010).

Regarding emotional gains, visual arts could promote personal growth and sense of purpose (Fisher and Specht 1999). Likewise, group musical activity could provide a connection to previous satisfying experiences (Wise et al. 1992). In addition, quality of life has been examined in relation to participation in numerous artistic disciplines, such as music, dance, theatre, literature and visual arts. A better quality of life was found in those who actively participate (Ho et al. 2019). Studies on participation in artistic activities highlight the benefits to well-being (Reynolds 2010), including spiritual well-being (Ho et al. 2019; Tzanidaki and Reynolds 2011).

Participation of older people in artistic activities could provide social benefits, such as interactions with other people through visual art-making (Fisher and Specht 1999), the establishment of social capital in choir participation (Reynolds 2015), and the construction of social status through craftwork (Tzanidaki and Reynolds 2011). This type of participation could involve a wide range of support and the sense of “family” (Reynolds 2015) and could act as a preventive measure against isolation (Maidment and Macfarlane 2011; Wise et al. 1992). Group experiences, such as participation in a community choir, may provide a sense of membership that allows older participants to be part of society (Southcott and Joseph 2015). Similarly, musical participation promotes the conservation and diffusion of heritage to the community (Joseph and Southcott 2018). The social gains obtained through artistic participation have been explored mainly in older amateur artists.

##### Negative consequences of artistic activities

Negative outcomes of artistic activities are a less studied topic. At individual level, music performance anxiety has been explored. It was found that older music performers recognized threats to their ego, which affected this specific type of anxiety. Nevertheless, even in this study, older musicians described more benefits of musical participation than negative aspects (Barbeau and Mantie 2019). Studies focused on dancing and specifically ballet found that injuries suffered by professional dancers could jeopardize their vocation for ballet and threaten their identity (Wainwright and Turner 2004). In terms of social elements, a professional performing career could produce limited financial resources and thus the need to sacrifice income, time and family. Following a performing arts career frequently means an unstable salary and irregular working hours (Mullen et al. 2012).

#### Experiences

Seventeen papers focused on older adults’ experiences of artistic participation. Papers about experiences cover issues associated with meanings attributed by the participants themselves, and events and dynamic aspects of artistic activities.

Some studies on arts and crafts participation experiences have focused on the meanings attached by older artists to creative practice (Hunt et al. 2018). Artistic activity could be perceived as a meaningful occupation, especially due to the feelings experienced during the activity (Pöllänen 2013), or as a medium for conserving and teaching arts and crafts tradition (Tzanidaki and Reynolds 2011). Other meanings could be related to the promotion of a “master identity” or a positive sense of self associated with care-giving relating to a person close to someone with dementia (Hunt et al. 2018). In musical participation, the meaning can be drawn from the sense of purpose in musical engagement (Matsunobu 2018).

Other papers explored artistic activities in the context of illness (Reynolds 2004). They found that artistic occupations represent a strong means of living positively with physical discomfort. Experiences of artistic activities have also been collected in the context of leisure activities. It is understood that these experiences are perceived in a way that promotes feelings of connectedness with the broader physical and social spheres (Reynolds 2010), and helps to construct an occupational identity (Howie et al. 2004), or reshape identity (Jenkins and Southcott 2016). The experiences of professional artists, differ from those of amateur artists: for example, how they adapt to the changes caused by aging, and the strategies they use to overcome these challenges and preserve their professional career (Barton 2004).

Moving on to another topic, spiritual elements are examined to a lesser extent. However, it seems that shared experiences as a leader of a musical group may be linked to spiritual elements, such as sense of gratitude to God or singing as a way to celebrate faith (Fung 2017). Regarding other areas, some papers have centred on creativity, showing how creative practice calls for a specific self-image focused on productivity (Gallistl 2018). Others seek to understand the relation between aging and creativity (Swinnen 2018), and look at writing as an art of living for older poets. Finally, certain papers focused on dynamic elements of artistic participation. This is the case of the study by Gamliel (2012), which aimed to confront the congruity of dramaturgical metaphors among older actors, and to look at representations of these metaphors. It was found that actors do not distinguish between performative self and interior self in their efforts to describe themselves.

## Discussion

This study aimed to critically examine the development of research on older adults’ participation in artistic activities. It was designed to explore which aspects of artistic activity in later life have been considered in studies, to identify knowledge gaps and to propose new directions for research.

Our scoping review shows that research on the topic has increased progressively over the last ten years. In particular, empirical studies have increased in this period. From the results of the review, we identified five main gaps that should be considered in future research about older people’s participation in artistic activities. These can be classified as related with: the artistic activities considered; the potential costs and barriers of older adults’ participation in artistic activities; older adults’ voices and their diversity; a life-course perspective; and a contextual view of research on older adults’ participation in artistic activities.

### Broadening the scope of artistic activities considered

First, through the scoping review, we observed that certain disciplines have been examined much more than others. This is the case of studies about older adults’ participation in musical activities, including singing (Joseph and Southcott 2017) and playing a musical instrument (Gembris and Heye 2014). Although this focus of research is an interesting, broad topic of study, other artistic disciplines such as photography, theatre, dance or literature are underrepresented. Reynolds et al. (2016) noticed this gap and the lack of literature on theatre and drama participation by older adults. In addition, more than a fifth of the papers in our study focused on two or more artistic disciplines. Although in some cases the reason for the inclusion of more than one discipline is justified, for example forms of arts and crafts (Reynolds 2009; Tzanidaki and Reynolds 2011), most empirical studies do not justify the inclusion of various artistic disciplines in the sample and do not compare art forms. This situation carries the risk of overgeneralizing research results, without considering the particular characteristics of artistic disciplines, for example, results may be generalized between performing arts and visual arts.

### Moving beyond benefits: Addressing potential costs and barriers for older adults’ participation in artistic activities

Second, three-quarters of the empirical papers are linked to the outcomes of older adults’ participation in artistic activities and specifically the benefits of these practices. The focus on the benefits of participation in artistic activities results in positive effects, such as the promotion of art as a meaningful activity, or the recognition of diverse ways to actively and successfully age. However, this “benefits focus” carries some risks. For example, a potential risk is to reinforce ageism and ableism (Martinson and Berridge 2015), forcing older people to be creative in all facets of life (Florida 2012). In addition, the focus on the positive consequences of artistic activities may introduce older people’s artistic participation in a biased way, as it does not explore the possible costs at personal, social or economic level of artistic participation, elements which have been largely neglected by research on this topic. A challenging theme for future research could be the effect, including potentially negative impacts, of an artistic career in other domains of the artist’s life, such as their family life or their partner relationship.

Similarly, research on the antecedents of artistic participation is predominantly focused on facilitators of these creative practices, to the detriment of the study of potential barriers. Understanding obstacles to older adults’ participation in artistic activities is vital to address artistic engagement and therefore to increase the benefits of these activities. In addition, knowledge of barriers created by cultural organizations and institutions could reduce these potential obstacles and ensure the artistic participation of older people. For example, some studies focused on professional artistic participation and explored the gender gap in the performing arts. The results showed that women are more likely than men to have less stable, lower paid employment. Moreover, this gender gap increases as performing women artists age (Coulangeon et al. 2005). Thus, an interesting topic for future research could be the intersection of gender and age in relation to creativity (Swinnen 2018).

### The absence of older adults’ voices and their diversity

Third, the predominance of studies on the benefits of older adults’ participation in artistic activities coexists with a lack of research about experiences. Lamb et al. (2017) point out that the voices of older people themselves are largely absent from existing research. The study of artistic experiences could support the exploration of the experience of aging and the meaning that people attach to later life, and the use of artistic activity for examining individual and social processes (Carr et al. 2009). The study of experiences of artists who need to adapt to the process of aging and renegotiate their connection to the successful aging paradigm could contribute relevant elements to the research field (Swinnen 2018).

Together with a lack of studies on experiences, the exploration of diversity in older people’s participation in artistic activities is another relevant aspect that is overlooked. In the last decade, some studies focused exclusively on older women participating in artistic activities. However, these types of studies are still insufficient. Other minority groups such as older people belonging to ethnic or racial minorities, older immigrants, older adults with disability, older members of the LGBT community, or the oldest-old people are underrepresented in this topic research, if not absent. As highlighted by Tzanidaki and Reynolds (2011), the meanings of participating in culturally traditional disciplines of art-making should be investigated further with persons in other rural and minority communities. Likewise, research needs to include male and female perceptions, as female perceptions are underrepresented in studies about creative occupations (Tzanidaki and Reynolds 2011). Thus, artistic participation research including diversity could help to create a more complex, comprehensive, realistic vision of older participants in artistic activities.

### The need for a life course perspective

Fourth, our scoping review shows that life course elements could influence positively or negatively older adults’ artistic participation (Gembris and Heye 2014). However, the inclusion of the life course approach is still scarce. This situation is reflected in the low number of studies (only three) that use a longitudinal research design (Lamont et al. 2018; Liddle et al. 2012; Manturzewska 1990). The use of longitudinal studies would allow researchers to explore changes in older artists over their life course. The inclusion of a life course perspective could enhance research on older adults’ artistic participation, which would allow us to explore age-related changes in artistic life; how attitudes, motivations and needs change over time; and health issues and strategies for coping (e.g., Gembris and Heye 2014). In fact, this scoping review highlights the absence of studies exploring the interconnections and influences between older artists (and their artistic careers) and individuals from other domains of their lives, such as the family or work. An examination from the perspective of “linked lives” could be an interesting contribution to this field of research (Elder 1998). Additionally, research on costs and barriers for this type of participation could be enriched by the use of a life course perspective.

### The relevance of a contextual view on research about older adults’ participation in artistic activities

Finally, the focus of the variables examined in the studies was largely on individual, interpersonal aspects of older participants in artistic activities. Studies including environmental elements have been mostly ignored. Furthermore, most studies included in our scoping review was conducted using USA and UK samples. Other countries were clearly underrepresented or even absent. A context-free perspective of older adults’ participation in artistic activities overlooks the role of culture and its influence on this type of participation. A deeper examination of the context in which artistic participation of older adults takes place is fundamental to understand artistic engagement in later life. Moreover, it would reveal opportunities or barriers linked to accessibility and the influence of neighbourhood and community on older adults’ participation in artistic activities. Just as Reynolds and Prior (2011) suggest, social and financial support might have had a considerable influence on the artistic occupations of the study participants. For this reason, further research with less advantaged artists is recommended. Similarly, to understand the significance of artistic engagement among older people, research about the patterns and contexts of cultural participation is necessary (Goulding 2018).

### Limitations and conclusions

Despite the potential contribution of this scoping review, it has some limitations that should be considered in the interpretation of the results. First, the study focuses on literature published in peer-reviewed journals. Consequently, potentially important studies published in other formats (e.g., books, chapters and conference proceedings) may have been omitted. A second limitation is language. This scoping review included studies published in English, which probably influenced the characteristic of the sample. The review excluded relevant research in the field that was written in other languages.

Notwithstanding the above limitations and to the best of our knowledge, the study provides the first scoping review of older adults’ participation in artistic activities. It makes a relevant, original, gerontological, art-focused contribution to the field of arts and aging research. The gaps detected by this study show the need for more research to deepen understanding of older adults’ participation in artistic activities. Our findings can offer guidance to academic, social and policy fields, and contribute to confronting negative, reductionist views of later life through older people’s artistic participation.
